# Discovery of endogenous nitroxyl as a new redox player in *Arabidopsis thaliana*

**DOI:** 10.1038/s41477-022-01301-z

**Published:** 2022-12-23

**Authors:** M. Arasimowicz-Jelonek, J. Floryszak-Wieczorek, S. Suarez, F. Doctorovich, E. Sobieszczuk-Nowicka, S. Bruce King, G. Milczarek, T. Rębiś, J. Gajewska, P. Jagodzik, M. Żywicki

**Affiliations:** 1 Department of Plant Ecophysiology, Adam Mickiewicz University, Poznań, Poland; 2 Department of Plant Physiology, Poznań University of Life Sciences, Poznań, Poland; 3 Departamento de Química Inorgánica, Analítica, y Química Física, Universidad de Buenos Aires, INQUIMAE-CONICET, Buenos Aires, Argentina; 4 Department of Plant Physiology, Adam Mickiewicz University, Poznań, Poland; 5 Department of Chemistry, Wake Forest University, Winston-Salem, NC USA; 6 Poznan University of Technology, Institute of Chemistry and Technical Electrochemistry, Poznan, Poland; 7 Department of Computational Biology, Institute of Molecular Biology and Biotechnology, Adam Mickiewicz University, Poznań, Poland

**Keywords:** Stress signalling, Plant signalling, Abiotic

## Abstract

Nitroxyl (HNO) is the one-electron reduced and protonated congener of nitric oxide (•NO), owning a distinct chemical profile. Based on real-time detection, we demonstrate that HNO is endogenously formed in *Arabidopsis*. Senescence and hypoxia induce shifts in the redox balance, triggering HNO decay or formation mediated by non-enzymatic •NO/HNO interconversion with cellular reductants. The stimuli-dependent HNO generation supports or competes with •NO signalling, depending on the local redox environment.

## Abstract (web summary)

Endogenous formation of nitroxyl has been discovered by real-time detection in plant cells. Evidence shows that nitroxyl has a specific accumulation pattern different from nitric oxide in response to senescence- and hypoxia-induced redox shifts.

## Main

Nitroxyl (HNO/NO^–^, also termed azanone and nitrosyl hydride) has been documented as a weak acid (p*K*_a_ 11.4), suggesting that HNO, rather than the nitroxyl anion (NO^–^), predominates at physiological pH^1,2^. The chemistry of this triatomic species is highly complex; HNO can react with many targets, including molecular oxygen, nitric oxide, nitrite, hydroxylamine, sulfite, thiosulfate, metalloproteins, metalloporphyrins, thiols, C- and S-nitroso compounds, nitroxides and phosphines^3,4^. Moreover, because metallo- and thiol-containing proteins are its main biological targets, HNO may also be a signalling molecule^5^.

On the other hand, the HNO relative nitric oxide (•NO) was identified as a physiological mediator of endothelial cell relaxation in mammalian systems in the late 1980s^6^, and has since proven to be a master regulator of numerous physiological and pathophysiological processes in all kingdoms. In land plants, •NO is synthesized endogenously via either reductive or oxidative routes^7^. Its generation fluctuates with various developmental and stress stimuli, tightly balanced by the formation of other reactive nitrogen species (RNS), highlighting •NO complex biology in cells^7,8^. Most of the impact of •NO in plants is attributed to its uncharged state or peroxynitrite. However, pharmacological studies in mammalian systems also emphasize the potential functionality of HNO^1,3^.

Despite intense research on HNO’s biological effects, its endogenous formation had not been detected in mammalian cells^9,10^, and in plants, HNO-generating systems and nitroxyl involvement in triggering biological responses were virtually terra incognita. Here, we present the first experimental evidence that HNO is formed endogenously in living cells of the model plant *Arabidopsis thaliana* (L.). Further, using precise methods to detect HNO and specific modulators of nitroxyl formation or scavenging, we show that the cellular redox environment tightly balances HNO formation.

In real time we used an electrochemical microsensor to measure HNO concentrations up to low nanomolar levels (1 nM–1 µM). Our previously described method is based on a three-electrode system consisting of a platinum counter electrode, an Ag/AgCl reference electrode and a gold working electrode modified with a cobalt porphyrin covalently attached via a thiol moiety^11,12^. Note that this method targets the HNO molecule; no interference or spurious signal arises from the presence of •NO, O_2_, NO_2_^−^ or other RNS^11–13^.

**Fig. 1:**
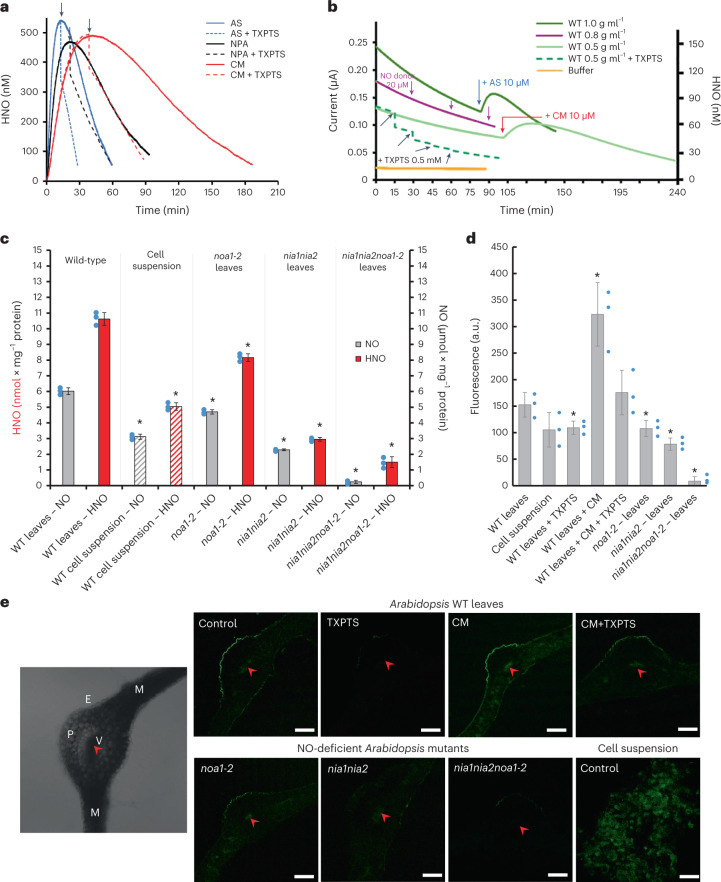
Detection of HNO in *Arabidopsis*. **a**, HNO concentration versus time, after adding 2 mM AS (blue), NPA (black) and CM (red), respectively, to a stabilized sensor baseline at 25 °C. The dotted line represents the addition of 1 mM TXPTS, a HNO scavenger, to each donor at the peak of HNO release. No signal was detected after adding 2 mM *S*-nitroso-*N*-acetylpenicillamine, a nitric oxide donor. **b**, Concentration/time traces of HNO endogenously generated in extracts from WT *Arabidopsis* leaves monitored by a nitroxyl biosensor at 25 °C. The concentration of leaf extracts is given in g ml^−1^. The dotted line represents when 0.5 mM TXPTS was added to 10 µM AS (blue arrow) or CM (red arrow; used as positive control). The black arrow indicates when phosphine was added (negative control) in all cases. No signal was detected after adding 20 μM *S*-nitroso-*N*-acetylpenicillamine (violet arrows). HNO and •NO sensors calibrations are presented in Extended Data Fig. 1. **c**, Endogenous levels of HNO and •NO in extracts from control WT leaves, cell suspension and *noa1-2*, *nia1nia2* and *nia1nia2noa1-2* NO-deficient mutant leaves. **d**, Fluorescence quantification in homogenates of leaf discs or cell suspension incubated with 16 μM phosphine-based fluorescent probe (excitation, 465 nm; emission, 520 nm). a.u., arbitrary units. **e**, Representative confocal laser scanning fluorescence microscopy images of a cross-section of *Arabidopsis* leaf or a cell suspension stained with 16 μM phosphine-based fluorescent probe (excitation, 488 nm; emission, 505–530 nm) in the absence (control) or presence of CM (1.5 mM), TXPTS (5 mM) or CM + TXPTS, respectively. Red arrows and V, vascular tissues; E, epidermis; P, parenchyma; M, mesophyll; scale bars, 100 μm; cell suspension, 40 μm. Bright-field images are presented in Extended Data Fig. 9a,b. For confocal observation, leaf cross-sections were randomly selected from ~20 slices pooled from leaves of different plants of the same genotype. The experiment was repeated independently three times with similar results. Data are presented as the mean ± s.d. of three biologically independent replicates (*n* = 3). *Values differ significantly (*P* ≤ 0.05) from control WT. Statistical significance was assessed using two-tailed *t*-tests. Source data

**Extended Data Fig. 1:**
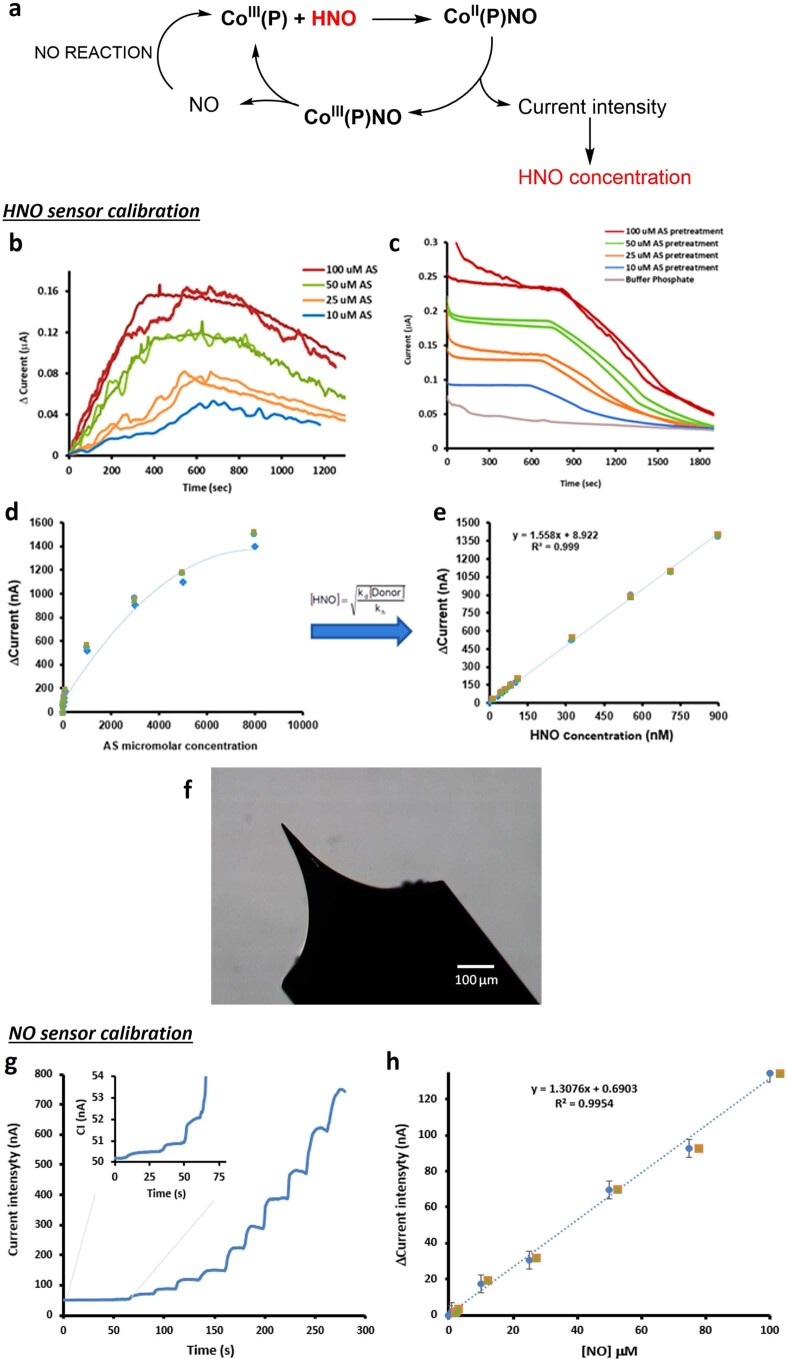
Nitroxyl (HNO) and nitric oxide (NO) biosensors calibration. **a**, Mechanism of specific electrochemical detection of HNO by cobalt porphyrin (CoP) covalently bonded to a gold surface. The developed sensor is an electrochemical HNO sensing device based on the covalent bonding of a cobalt porphyrin to a gold surface. A surface effect modulates the redox potentials allowing discrimination between HNO and NO. The electrode potential is set at a value of 0.8 V, where the porphyrin is stable as Co(III)P, observing a basal current. The reaction with HNO produces the Co(III)PNO¯ complex, which under the conditions described above is oxidized to Co(III)PNO. Due to the lability of the resulting Co(III)PNO complex, the system rapidly returns to Co(III)P, allowing the catalytic cycle to begin again. Since each electrode is only covered with a surface concentration, the total amount of trapped HNO is very small. Also, it is important to note that since a small amount of current can be detected, the ‘real’ amount of azanone reacting with the electrode is generally negligible compared to the total amount of HNO in the system (less than 1 %), and does not significantly perturb the production and consumption of HNO, making it a powerful tool in mechanism and kinetics studies where azanone plays a key role.^11,12^ **b**-**e**, HNO biosensor calibration; **b**, Current response after adding different Angeli’s salt (AS) concentrations to a stabilized sensor’s baseline. One microliter of an AS stock solution was added to a stabilized sensor’s baseline. A few seconds after, the signal starts to grow, and after 15 min, it begins to decrease. No signal was detected after adding SNAP, a nitric oxide donor. **c**, Current response after pretreatment of 10 min of different AS concentrations. One microliter of an AS stock solution was added to 1 ml of buffer and then was waited for 10 min. Next, the measurement with the sensor was started. In this case, as expected, the signal only decreased. **d**, Δcurrent vs. AS concentration. **e**, Δcurrent vs. HNO concentration. The HNO concentration in the Co(P) electrode reaction solution was estimated by the following kinetic analysis^11,12^: $Donor\,\left( {AS/PA} \right)\mathop{\longrightarrow}\limits^{{k_d{\it{v}}_d}}HNO$ (1) $2HNO\mathop{\longrightarrow}\limits^{{k_{h,}{\it{v}}_{\it{h}}}}N_2O + H_2O$ (2) Where reaction (1) represents the donor decomposition characterized by a first-order rate constant k_d_, and (2) represents bimolecular HNO dimerization, characterized by rate constant k_h_. Reaction (1) was considered an irreversible reaction because its inverse rate is negligible concerning reactions (2) at the initial rate condition. Reaction (2) is irreversible. Calculating [HNO], all the HNO produced through reaction (1) must be consumed in reaction (2), therefore both rates must be equal: *v*_*d*_ = *v*_*h*_. The donor decomposition obeys first-order kinetics, characterized by *k*_*d*_(*k*_*d(AS)*_ = 8 ×10^−4^ s^−1^)^14,58^ while the dimerization is a second-order rate reaction characterized by *k*_*h*_ (k_*h*_ = 8 ×10^6^ M^−1^s^−1^)^2,59,60^, thus $k_{d(AS)}\left[ {AS} \right] = k_h\left[ {HNO} \right]^2$(3) therefore [HNO] can be estimated as: $\left[ {HNO} \right] = \sqrt {\frac{{k_d\left[ {Donor} \right]}}{{k_h}}}$(4) **f**, Micrograph of Au tip microelectrode fabricated by electrochemical etching under constant potential of 1.3 V vs. Ag/AgCl in 2.8 M KCl. The gold wire served as working electrode, platinum ring was applied as counter electrode and Ag/AgCl (3 M KCl) was a reference electrode. The applied constant potential to the Au working electrode was 1.3 V. Supporting electrolyte was 2.8 M KCl. The gold wire was immersed into the solution to *ca*. 1 mm in depth. The effect of electrochemical etching was assessed microscopically on each microelectrode fabrication. **g**-**h**, NO sensor calibration. **g**, Response of the sensor to increasing concentration of NO, generated *in situ* from the reaction of iodide with nitrite in acid solution^41^. No signal was detected after adding a nitroxyl donor. **h**, Relationship between NO concentration and electrode current measured. Data are presented as mean ± s.d. of three biologically independent replicates (*n* = 3). Source data

More specifically, we first verified the effectiveness of the microsensor’s HNO electrodetection in three structurally distinct donor solutions: Angeli’s salt (AS^14^), 4-NO_2_-Piloty’s acid (NPA^15^) and Cimlanod (CM, formerly BMS-986231 or CXL-1427 (ref. 16)) in the presence or absence of the HNO scavenger, phosphine tris(4,6-dimethyl-3-sulfonatophenyl)phosphine trisodium salt hydrate (TXPTS; Fig. 1a and Supplementary Table 1). Next, we recorded concentration/time traces of endogenous HNO generation in extracts from 21-day-old, wild-type (WT) *Arabidopsis* leaves (Fig. 1b). We detected a high current reaching 0.240 ± 0.005 µA and calibrated it against one measured in a known concentration of HNO in solution; both were 150 ± 3 nM (Extended Data Fig. 1).

**Extended Data Fig. 2:**
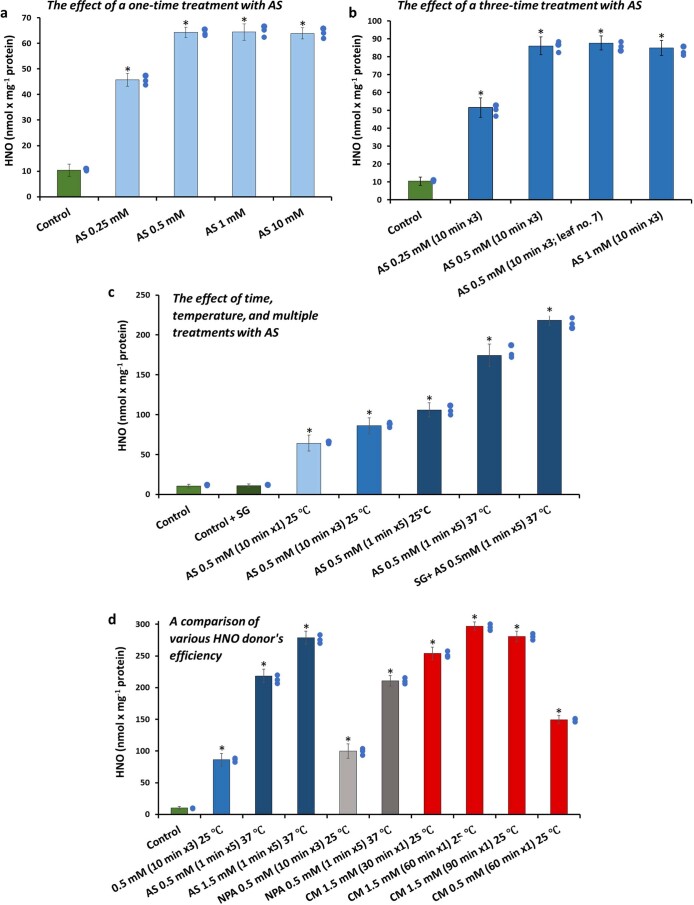
The treatment effect of Arabidopsis WT leaves with nitroxyl donors on HNO concentration. **a**, The impact of a one-time treatment of Arabidopsis WT leaves with Angeli’s salt (AS) on HNO concentration. Leaves were sprayed with the following AS concentrations 0.25, 0.5, 1.0, and 10 mM, respectively, at 25 °C. HNO measurements with microsensor were carried out in the leaf extracts 10 min after spraying. HNO formation was comparable in AS concentration range of 0.5–10 mM indicating a leaf penetration limit for AS diffusion. This could be explained by the fact that plant epidermis is covered by an extracellular hydrophobic layer (cuticles), and AS is the sodium trioxodinitrate salt (Na_2_[N2O_3_]). This difference between nonpolar and polar compounds, especially during multiple treatments, was optimized using an organic surfactant (see **c**). The treatment with the lowest AS concentration (0.25 mM) decreased (20%) the HNO level. **b**, The effect of a three-time treatment of Arabidopsis WT leaves with AS on HNO concentration. Leaves were three-time sprayed in 10 min intervals with the following AS concentrations 0.25, 0.5, 1.0 mM, respectively, at 25 °C. HNO measurements with microsensor were carried out in the leaf extracts 10 min after the third spraying The maximum HNO concentration in leaf tissues three-time treated with 0.5 mM AS increased by 40% compared with one-time pretreatment (for comparison see A). HNO concentrations measured in a group of leaves no. 5–7 or only leaf no. 7 (pooled from several plants) treated with AS (0.5 mM) were comparable. **c**, The effect of time, temperature, and multiple treatments of Arabidopsis WT leaves with AS on HNO concentration. Leaves were sprayed once, three, or five- times in 1 or 10 min intervals, respectively, with 0.5 mM AS at 25 °C or 37 °C. HNO measurements with microsensor were carried out in the leaf extracts after 1 or 10 min after the last spraying, respectively. Angeli’s salt decomposition rate is temperature-dependent, so HNO production from 0.5 mM AS was almost 2-fold lower at 25 °C than at 37 °C, respectively. **d**, A comparison of various HNO donor’s efficiency. Leaves were sprayed with AS, 4-NO_2_-Piloty´s acid (NPA), and Cimlanod (CM) at 25 °C or 37 °C. HNO measurement with microsensor was carried out in the leaf extracts after 5, 30, 60, or 90 min, respectively. The most stable and effective HNO donating compound turned out to be CM (1.5 mM) with stabilized HNO emission in 60–90 min, after once leaf treatment at 25 °C. A similar amount of HNO released AS (1.5 mM) in a short time after five-time leaf treatment with this donor at 37 °C. Both NPA and AS at lower concentrations (0.5 mM) produced less HNO due to the rapid rate of donors decomposition. For multiple treatments, an organic surfactant (0.1% SG) to improve the surface contact between the donor solution and the leaf was applied, as presented in Extended Data Fig. 2c. The crude extracts were prepared as described in the Methods. Data are presented as mean ± s.d. of three biologically independent replicates (*n* = 3). (*) indicate values that differ significantly (*P* ≤ 0.05) from control WT. Statistical significance was assessed using two-tailed *t*-tests. Source data

**Extended Data Fig. 3:**
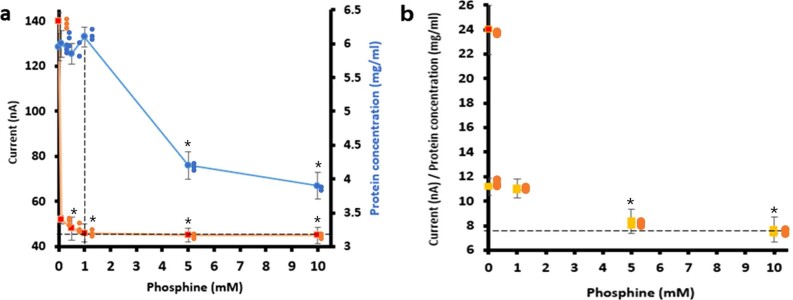
Phosphine (TXPTS) treatment mutes the HNO-dependent electrochemical signal. Two different approaches were performed to evidence that TXPTS traps HNO in plant systems including *in vitro* and *in vivo* treatment. **a**, In *in vitro* experiments, when several TXPTS concentrations were added directly to Arabidopsis WT extract (the crude extract was prepared as described in the Experimental Procedures). The graph a, presents current intensity (proportional to HNO concentration, red square, left axis) and protein concentration (blue circles, right axis) vs. phosphine TXPTS concentration (0, 0.1, 0.5, 1, 5 and 10 mM). **b**, In *in vivo* experiments, when Arabidopsis WT leaves were sprayed with TXPTS and incubated for 30 min at room temperature. The graph b, presents current intensity (nA) per protein concentration (mg/ml) without (red square) and after (orange squares) after a 30-min incubation of leaves with TXPTS (0, 1, 5, and 10 mM). In these experiments, protein concentration per gram of FW remained constant throughout all experiments (*n* = 10), with a value of 6.0 ± 1.2 mg/ml. After the addition of TXPTS, nitroxyl concentration decreases. It agrees with the selective detection of HNO since phosphines do not react with nitrite, nitrate, and NO donors at ambient temperature and physiological pH^61^. However, in *in vitro* experiment, protein concentration starts to decrease after adding 1 mM of phosphine. So, the best condition to perform *in vitro* HNO trapping experiments is to add at most 0.5 mM of TXPTS. Moreover, after the higher addition of phosphine, the electronic signal remains constant at 45 nA. This current intensity was used as the basal line for all experiments in this work. Figure b shows that leaves treatment with 5 mM of TXPTS was the most efficient dose in *in vivo* HNO trapping. Then the electronic signal per protein concentration after a half-hour was at 7.8 nA/(mg/ml). This parameter was used as the basal line for all experiments. Data are presented as mean ± s.d. of three biologically independent replicates (*n* = 3). (*) indicate values that differ significantly (*P* ≤ 0.05) from control (untreated) WT leaves. Statistical significance was assessed using two-tailed *t*-tests. Source data

To confirm that the sensor selectively detects HNO in the plant cellular environment under in vitro conditions, we added donors (10 µM AS or 10 µM CM) to release the molecule at physiological pH (Fig. 1b). Leaves pretreated with HNO-releasing agents (AS, NPA, CM) showed significant nitroxyl enrichment, but the electrochemical signal was dependent on donor type, concentration, time and temperature (Extended Data Fig. 2). To prove the presence of HNO in plant tissues, we selected a specific concentration of the water-soluble TXPTS as the trapping agent because it does not produce nitroxyl in reacting with other components of the extract media, especially NO and thiols (Extended Data Fig. 3).

Results confirm that under physiological (non-stress) conditions, the plant endogenously produces low (nanomolar) concentrations of HNO. The fainter amperometric signal reflecting a lower HNO concentration was detected in both WT *Arabidopsis* cell suspension and leaves of *Arabidopsis* mutants with a lower •NO content—*noa1-2* and *nia1nia2* (Fig. 1c). These results demonstrate that endogenous •NO determines HNO content in biological systems, possibly via nitric oxide one-electron reduction to nitroxyl^4^. Plant cells probably have other chemical sources of HNO because in the triple-mutant *nia1nia2noa1-2* we detected only trace amounts of •NO, whereas HNO content constituted over 10% of the value recorded in WT plants (Fig. 1c).

**Extended Data Fig. 4:**
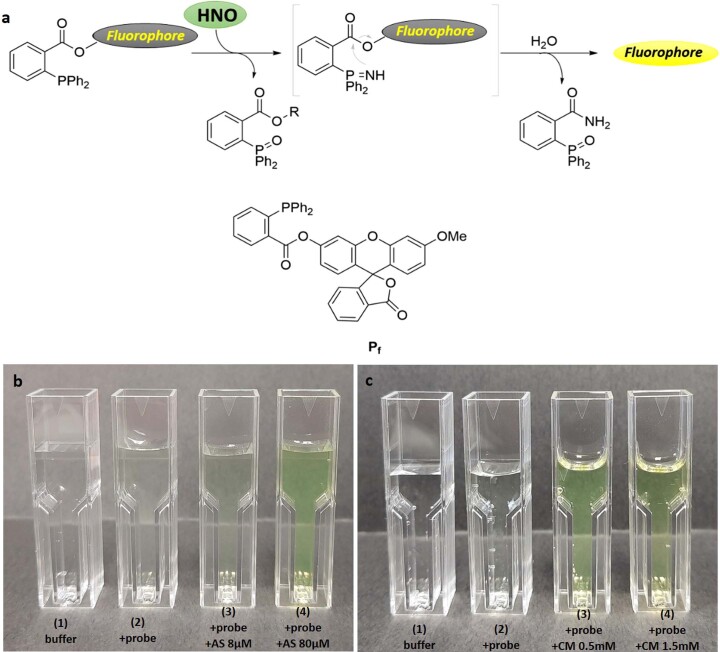
Fluorescence response of phosphine-based fluorescent probe after 2 h of incubation with HNO donors. **a**, Mechanism of HNO trapping by the phosphine-based fluorescent probe. Pf - fluorescent phosphine probe. The yellow-green color indicates HNO trapping by the probe. **b**, From the left: (1) control - buffer 10 mM Tris-HCl, pH 7.4; (2) probe in 10 mM Tris-HCl, pH 7.4; (3) 8 µM AS+ probe in 10 mM Tris-HCl, pH 7.4; (4) 80 µM AS+ probe in 10 mM Tris-HCl, pH 7.4. **c**, From the left: (1) control - buffer 10 mM Tris-HCl, pH 7.4; (2) probe in 10 mM Tris-HCl, pH 7.4; (3) 0.5 mM CM+ probe in 10 mM Tris-HCl, pH 7.4; (4) 1.5 mM CM+ probe in 10 mM Tris-HCl, pH 7.4.

To track HNO formation in plant cells, we used a fluorescent triarylphosphine-based probe that relies on the reductive Staudinger ligation of HNO with an aromatic phosphine^17^ (Extended Data Fig. 4a). By confirming the fluorophore’s ability to detect and estimate the HNO concentration in the plant cellular environment we performed quantification and bio-imaging of HNO in vivo in *Arabidopsis* leaves and a cell suspension (Fig. 1d,e and Extended Data Fig. 4b,c). Epidermal and leaf vascular bundles showed faint intracellular fluorescence. Adding CM enriched HNO production to enhance the signal. Co-treating the leaves with the scavenger TXPTS reduced, but did not quench, HNO-dependent fluorescence (Fig. 1d,e).

**Extended Data Fig. 5:**
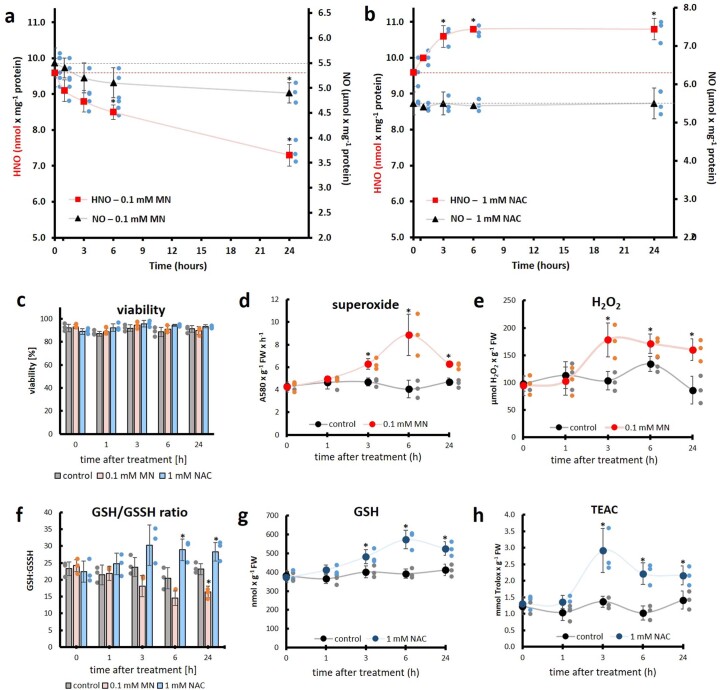
Kinetics of endogenous HNO and •NO formation under different cellular redox states in WT Arabidopsis leaves. **a**, The effect of a menadione-induced (0.1 mM MN) redox environment on HNO/•NO generation. **b**, The effect of an N-acetylcysteine-induced (1 mM NAC) redox environment on HNO/•NO generation. **c**, Neither redox modulator significantly changed cell viability of Arabidopsis WT leaves. Leaves treatment with 0.1 mM MN shifted the cellular environment toward oxidative, manifested by a reduced GSH/GSSH ratio, an elevated level of hydrogen peroxide, and superoxide (**d-f**). Leaves treatment with 1 mM NAC shifted the cellular environment toward reductive by enhanced GSH/GSSH ratio, the elevated pool of reduced glutathione, and an improved total antioxidant capacity (**f-h**). Data are presented as mean ± s.d. of three biologically independent replicates (*n* = 3). (*) indicate values that differ significantly (*P* ≤ 0.05) from control (untreated) WT leaves. Statistical significance was assessed using two-tailed *t*-tests. Source data

To determine whether the cellular redox status tightly regulates in vivo HNO formation, we shifted the redox balance in WT *Arabidopsis* leaves towards an oxidative or reductive environment by applying 0.1 mM menadione (MN) or 1 mM *N*-acetylcysteine (NAC), respectively (Extended Data Fig. 5). Electrochemical quantification revealed that MN pretreatment decreased the HNO level by ~20% and NAC pretreatment showed the opposite effect: the nitroxyl level increased by ~15% (Extended Data Fig. 5a,b).

**Fig. 2:**
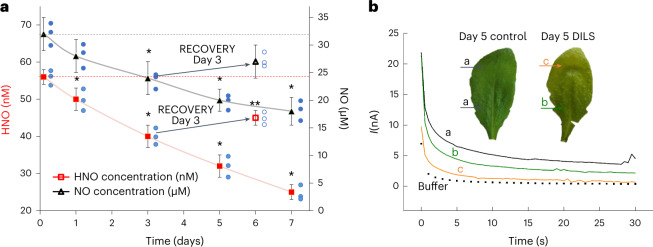
Kinetics of endogenous HNO formation in WT *Arabidopsis* plants during DILS. **a**, Electrochemical detection of HNO/•NO in leaves undergoing DILS. Black arrows indicate a 3-day recovery phase by reversing the DILS programme by restoring light access on day 3. **b**, HNO peak current recorded in vivo as a function of time. Letters (a, b, c) indicate leaf zones with different chlorophyll content (Supplementary Table 2). Data are presented as the mean ± s.d. of three biologically independent replicates (*n* = 3). *Values differ significantly (*P* ≤ 0.05) from day 0 of DILS, or day 3 in the case of recovery experiment. Statistical significance was assessed using two-tailed *t*-tests. Source data

**Extended Data Fig. 6:**
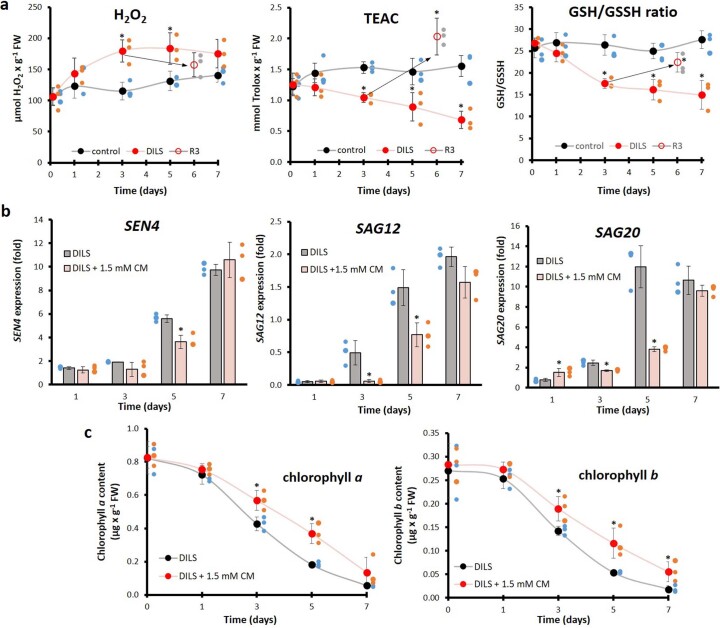
Nitroxyl implications in dark-induced leaf senescence (DILS) of WT Arabidopsis. **a**, Redox parameters of leaf cells undergoing DILS. H_2_O_2_, hydrogen peroxide; TEAC, total antioxidant activity; GSH/GSSH ratio, the ratio of reduced to oxidized glutathione. Black arrows indicate a three-day recovery phase by reversing the DILS program by restoring light access on day 3. **b**, The effect of applying exogenous HNO as 1.5 mM CM on the expression of senescence-associated genes (*SAGs*), *SAG12*, *SAG20*, and *SEN4*, which are molecular markers of the changes underpinning senescence^62^. **c**, Chlorophyll content in leaves undergoing DILS. Data are presented as mean ± s.d. of three biologically independent replicates (*n* = 3). (*) indicates values that differ significantly (*P* ≤ 0.05) from (a) control leaves or day 3 of DILS in case of recovery experiment, or (b, c) untreated leaves undergoing DILS. Statistical significance was assessed using two-tailed *t*-tests. Source data

Based on our observations, we hypothesized that developmental or environmental stimuli alter the cellular redox balance and provoke HNO fluctuations. To test this assumption, we subjected WT *Arabidopsis* plants to dark-induced leaf senescence (DILS) as a model for the perturbation of redox homeostasis^18^. As expected, senescence promoted an unfavourable oxidative environment for HNO (Extended Data Fig. 6a). Electrodetection in extracts of individually darkened WT *Arabidopsis* leaves (leaf seven of the rosette) revealed a significant (~50%) decrease in HNO signal from day 1 to day 7 (Fig. 2a). In vivo, a needle-type electrode (Extended Data Fig. 1f) indicated that HNO-dependent current was weakest in the leaf zone where chlorophyll content decreased most sharply (zone c in Fig. 2b and Supplementary Table 2). Notably, reversing the DILS programme by restoring light access on day 3 recovered the HNO pool and increased chlorophyll content (Fig. 2a and Supplementary Table 2). Nitroxyl-pretreated leaves showed significantly less accumulation of senescence-associated gene transcripts, including *SAG12* encoding cysteine protease, and less chlorophyll degradation, indicating that from day 3, senescence was delayed (Extended Data Fig. 6b,c). We conclude that fluctuation in HNO production is a part of the redox pathway that modulates dark-induced senescence.

**Fig. 3:**
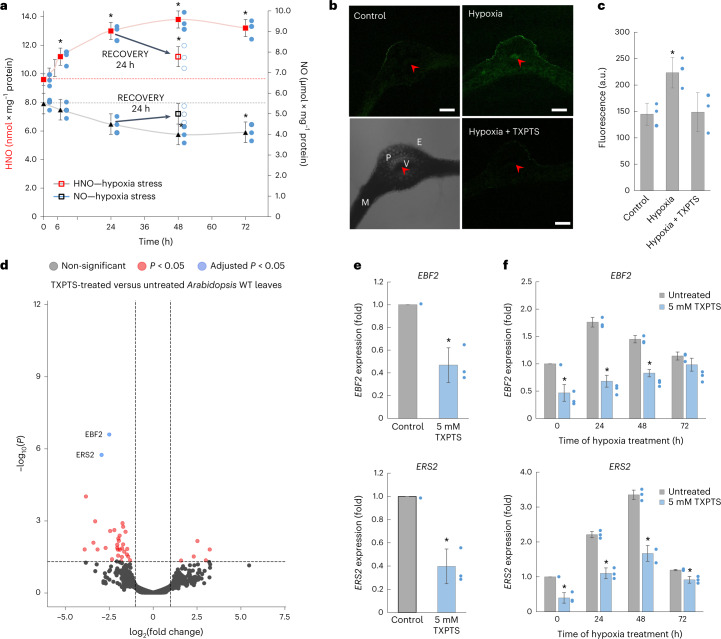
Kinetics of endogenous HNO formation in WT *Arabidopsis* leaves exposed to hypoxia and identification of HNO-responsive genes. **a**, Electrochemical detection of HNO/•NO during hypoxia. Black arrows indicate a 24 h recovery phase by restoring normoxia conditions after 24 h of hypoxia. **b**, Representative confocal laser scanning fluorescence microscopy images of a cross-section of *Arabidopsis* leaf stained with 16 μM phosphine-based fluorescent probe (excitation, 488 nm; emission, 505–530 nm) in the absence or presence of TXPTS (5 mM) at 48 h of hypoxia. Red arrows and V, vascular tissues; E, epidermis; P, parenchyma; M, mesophyll; scale bars, 100 μm; bright-field images are presented in Extended Data Fig. 9c. For confocal observation leaf cross-sections were randomly selected from ~20 slices pooled from leaves of different plants of the same genotype. The experiment was repeated independently three times with similar results. **c**, Fluorescence quantification in homogenates of leaf discs incubated with 16 μM phosphine-based fluorescent probe (excitation, 465 nm; emission, 520 nm). **d**, Volcano plot of the differential gene expression results from the comparison of TXPTS-treated versus control (untreated) samples. The *x* axis represents the log_2_(fold change) of expression, the *y* axis represents the statistical significance of the expression change (*P*-value). Adjusted *P*-values for differentially expressed genes were derived from two-sided limma test, subjected to Benjamini-Hochberg correction. Statistically significant genes are shown in colour according to the legend. **e**, Validation of differentially expressed genes in response to TXPTS treatment using quantitative polymerase chain reaction. **f**, Expression of the identified HNO-responsive genes during hypoxia. Data are presented as the mean ± s.d. of three biologically independent replicates (*n* = 3). *Values differ significantly (*P* ≤ 0.05) from (**a**) 0 h time point or the 24 h time point in the case of recovery experiment; (**c**,**e**,**f**) control untreated leaves. Statistical significance was assessed using two-tailed *t*-tests. Source data

To further support our hypothesis that cellular redox status is decisive for HNO kinetics, WT *Arabidopsis* plants experienced hypoxia associated with reductive conditions. Hypoxia encouraged a significant (~25%) increase in HNO formation mainly during the first 24 h (Fig. 3a–c). One day after the stress was removed, HNO decreased sharply (Fig. 3a). These results indicate that a switch in nitroxyl kinetics toward HNO formation is an early reductive stress-related response in plant cells. Because hypoxia can also occur in plant tissues and organs under normoxia^19^, it creates (micro)hot-spots of HNO bioavailability and bioactivity.

**Extended Data Fig. 7:**
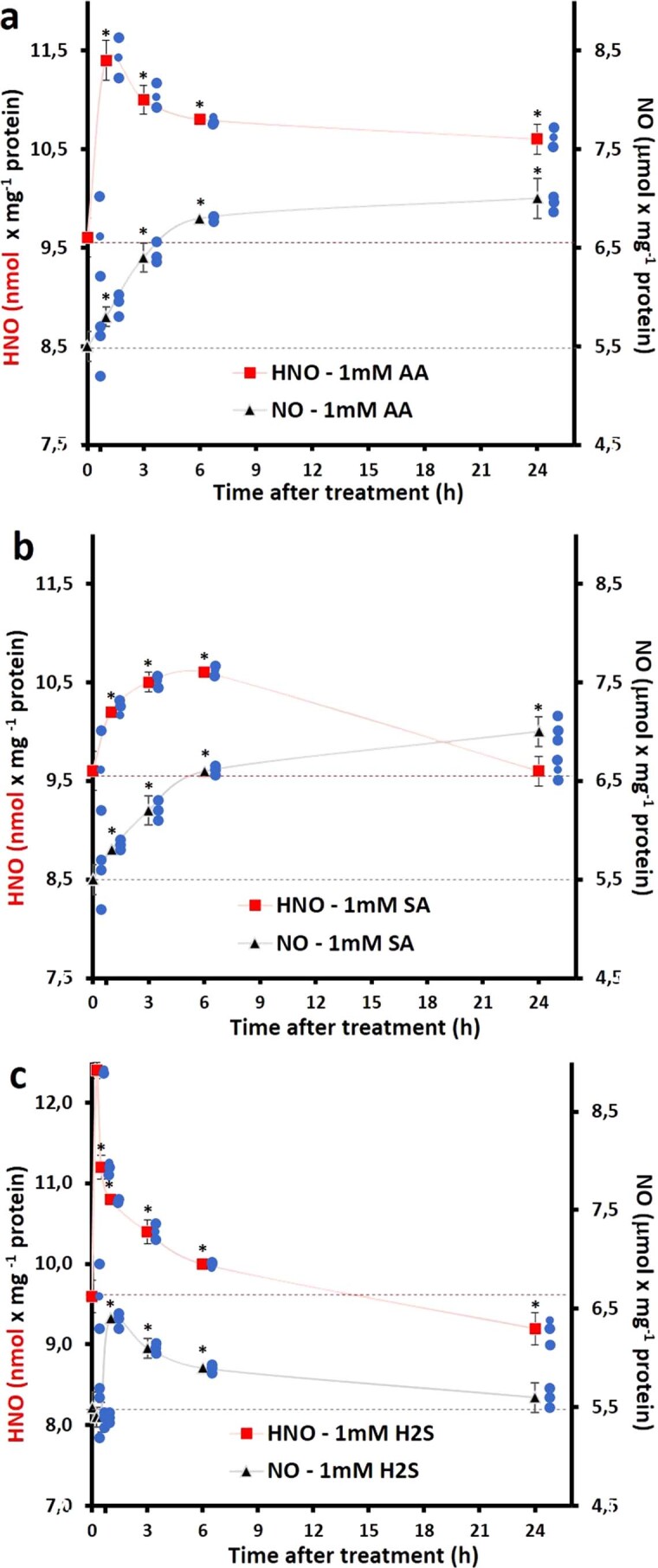
Kinetics of endogenous HNO and •NO formation in WT Arabidopsis leaves pretreated with cellular reductants. **a**, The effect of reducing conditions induced in leaves by ascorbic acid (1 mM AA), b, salicylic acid (1 mM SA) and c, hydrogen sulphide (1 mM H_2_S) on HNO/•NO generation. Enrichment of leaves with pseudo-aromatic alcohols such as AA and SA (**a, b**) resulted in a time-dependent rise in the HNO level measured *in vivo* by electrodetection in leaf extracts. However, ascorbate as a diol may react *in vitro* approximately twenty times faster than SA being a phenol^63,64^. The proposed mechanism involves a nucleophilic attack to •NO by the alcohols, coupled with proton transfer and subsequent decomposition of the thus-produced radical to yield HNO (Supplementary Table 3). Leaf pretreatment with H_2_S (**c**) also resulted in a significant increase in HNO production. The signal accelerated during the first minutes reflecting the fast nature of the reaction between H_2_S and •NO to produce HNO (k = 10^4^ M^−1^s^−1^)^65^. However, there is a more complicated mechanism than a simple bimolecular reaction between •NO and H_2_S/HS^−^ (Supplementary Table 3), but the response of the successful attack by two NO molecules to this gasotransmitter (H_2_S) produces HNO^4,65^. In this context the reported redox potential, E´(NO, H^+^/ HNO), at physiological •NO and HNO concentration is between −0.3 and 0 V at pH = 7.4. Thus, the one-electron reduction process for NO is feasible under biological conditions and could be promoted by the analyzed reductants with reduction potentials around −0.3 to −0.5V^4,65^. Data are presented as mean ± s.d. of three biologically independent replicates (*n* = 3). (*) indicate values that differ significantly (*P* ≤ 0.05) from control (untreated) WT leaves. Statistical significance was assessed using two-tailed *t*-tests. Source data

Searching for physiologically relevant HNO sources under a reductive environment, we also confirmed that non-enzymatic •NO/HNO interconversion mediated by cellular reductants such as ascorbic acid, salicylic acid or hydrogen sulfide (H_2_S)^4^ might constitute an important in vivo route to HNO formation in plant cells. Enrichment of leaves with pseudo-aromatic alcohols and H_2_S resulted in a time-dependent rise in the HNO level (Extended Data Fig. 7 and Supplementary Table 3). All the three compounds are essential players in plant cells involved, for example, in signal transduction at physiological concentrations from µM (H_2_S, salicylic acid) to mM (ascorbic acid)^20–22^ and may provide ubiquitous HNO bioavailability.

**Extended Data Fig. 8:**
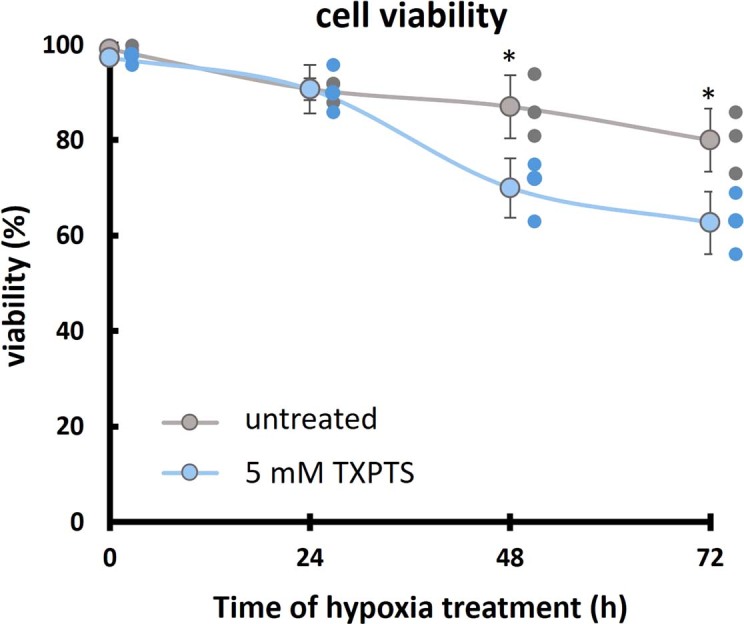
The effect of HNO scavenger treatment on cell viability of Arabidopsis WT leaves undergoing hypoxia. Rosette Arabidopsis leaves were sprayed with the HNO scavenger, TXPTS (5 mM) or distilled water (untreated) and exposed to hypoxia stress as described in Methods. Data are presented as mean ± s.d. of three biologically independent replicates (*n* = 3). (*) indicate values that differ significantly (*P* ≤ 0.05) from control (untreated) WT leaves. Statistical significance was assessed using two-tailed *t*-tests. Source data

Finally, to discover the physiological significance of the redox-dependent HNO fluctuations in WT *Arabidopsis* leaves, profiling of the HNO-dependent transcriptome (TXPTS-treated) was performed. The functional analysis of gene set enrichment revealed that the most significantly enriched Gene Ontology (GO) categories were ethylene receptor activity (molecular function, GO:0038199) and negative regulation of the ethylene-activated signalling pathway (biological process, GO:0010105). Of all the above genes, two met the strict criteria for the significance of differential expression with the adjusted *P*-value <0.05: EIN3-binding F-box protein 2 (EBF2 – AT5G25350) and ethylene response sensor 2 (ERS2 – AT1G04310; Fig. 3d and Supplementary Table 4). The observed implication of HNO in regulation of the ethylene response could be critical for responses to environmental changes with elevated HNO formation. ERS2 belongs to a group of membrane-located receptor proteins, whereas EBF2 governs ethylene signalling diminishing and/or resetting ethylene responses in cells with initiated transduction of the hormone signal^23^. In confirmation, hypoxia-induced HNO generation coincides with *EBF2* and *ERS2* upregulation mainly in the first 48 h of the stress. No change or downregulation of *EBF2* and *ERS2* expression coinciding with significantly reduced cell viability was observed when TXPTS trapped HNO during hypoxia stress (Fig. 3e,f and Extended Data Fig. 8). The results shed light on potential HNO implication in a well proved NO/ethylene signalling pathway for example Hartman et al.^24^

These studies are the first to confirm endogenous HNO production in living cells and point toward a novel regulatory role of the molecule in the ethylene signalling pathway in plants. Added to our previous findings on HNO chemistry^4,11,13,15^, our revelation of HNO’s effects on plant •NO signalling and metabolism mandate the inclusion of nitroxyl in the group of gasotransmitters now composed of •NO, CO and H_2_S. The ubiquitous bioavailability of the HNO molecule provided by non-enzymatic •NO/HNO interconversion allows it to support or compete with •NO signalling, depending on the local redox environment. Nitroxyl’s contribution to the reactive species interactome engaged in cell signalling requires re-evaluation of the consensus on •NO biology. Our results provide the impetus and a scaffold to explore the biological functions of HNO in living cells.

## Methods

### Plant material and growth conditions

The Columbia (Col-0) ecotype of *Arabidopsis thaliana* was used as the WT. T-DNA insertion lines, *nia1nia2* (SALK_ N2356) and *noa1-2* (SAIL_507_E11) were obtained from the SIGnAL collection of the Nottingham Arabidopsis Stock Centre^25^. The triple *nia1nia2noa1-2* mutant (impaired in nitrate reductase and nitric oxide-associated 1 (NOA1)-mediated NO biosynthetic pathways) was generated by crossing, and the putative triple homozygous mutant plants were confirmed by polymerase chain reaction (PCR) and sequence analyses, following Lozano-Juste and León^26^. The PCR primers (Genomed) used for genotyping SALK lines and triple mutants are listed in Supplementary Table 5.

After 3 d of cold stratification, all seeds were planted in a growth chamber under a 16:8 h light/dark cycle at a photon fluency rate of 110 μmol m^−2^ s^−1^ at 22 °C and 60% relative humidity. *nia1nia2noa1-2* was cultivated with Murashige and Skoog medium supplemented with nitrites to promote growth. The experiments were performed on leaves from 21-day-old plants.

*Arabidopsis thaliana* (Col-0) cell suspension cultures from leaf-derived callus were grown following Encina et al.^27^ in a modified Murashige and Skoog^28^ medium (including vitamins, Duchefa Biochemie) enriched with 30 g l^−1^ sucrose. They were subcultured every 21 d when 2 ml were transferred into 50 ml of fresh medium in 500 ml flasks. The cultures were grown on a shaker at 120 rpm under a 16:8 h light/dark cycle at a photon fluency rate of 110 μmol m^−2 ^s^−1^ at 22 °C. Experiments were carried out on d21 of the cultivation cycle.

### DILS

To initiate DILS, rosette leaf seven from a 21-day-old WT *Arabidopsis* plant was darkened by gently covering it with aluminium foil; the rest of the rosette remained under the normal light/dark cycle. Senescence progression was monitored on d1, d3, d5 and d7. Control (non-darkened) samples were also harvested from leaf seven.

### Hypoxia stress and recovery procedure

In hypoxia treatments, 21-day-old WT plants were subjected to a low-O_2_ air mix in an airtight chamber under the same light/dark cycle. Air-flux conditions were 3% O_2_, 0.03% CO_2_ and 97% N_2_ gas. Control plants were maintained at a normal oxygen level (normoxia). Analyses were performed 1, 3, 6, 12, 24, 48 and 72 h after treatment. One group of plants was removed from the chamber at 24 h and recovered under normal growth conditions for 24 h.

### HNO-modulator treatments

To test the efficiency of our modulators (Extended Data Figs. 2 and 3) at enriching or quenching HNO in plant tissues, rosette *Arabidopsis* leaves were sprayed with different concentrations of HNO donors, such as AS (0.25, 0.5, 1, 1.5 and 10 mM), NPA (0.5 mM) and CM (0.5 and 1.5 mM) (MedChemExpress), or the HNO scavenger TXPTS (0, 1, 5 and 10 mM) and incubated under various time and temperature conditions (Extended Data Figs. 2 and 3) in an airtight chamber. For electrochemical measurements of HNO concentration, a crude extract was prepared from leaves five to seven as described in the section ‘Crude leaf extract preparation for electrochemical measurements’.

To study the effect of CM on physiological and molecular parameters during DILS, the seventh leaf of the rosette of the 21-day-old plants was sprayed once with 1.5 mM CM (MedChemExpress), incubated for 1 h in an airtight chamber, then subjected to DILS as described above. Control plants, also collected from leaf seven, were treated with distilled water and incubated for 1 h in an airtight chamber. To study the effect of TXPTS on transcriptomic changes and physiological parameters, leaves of the rosette of 21-day-old plants were sprayed once with 5 mM TXPTS and incubated for 2 h in an airtight chamber under normal growth conditions; control plants were treated with distilled water, and incubated as above. All collected samples were immediately used for experiments (chlorophyll measurement) or frozen in liquid nitrogen and stored at −80 °C until further use (RNA extraction). Each sample consisted of three leaves (leaf seven) pooled from different plants.

### HNO quantification by electrochemical method

A three-electrode system consisting of a platinum counter electrode, Ag/AgCl quasi-reference electrode and a gold working electrode modified with a cobalt porphyrin covalently attached via a thiol moiety was used to detect HNO, following a previously described method^11,12,29,30^. Briefly, in the presence of HNO, a CoIII(P)NO-adduct forms and oxidizes. The resulting CoIII(P)NO is unstable, thus completing the catalytic cycle. The current intensity is proportional to the amount of HNO that binds to Co(P)^12^ (Extended Data Fig. 1a). TEQ_HNO software v.2.0 was used for HNO data collection.

For in vivo measurement, electrochemical etching was used to form the gold working electrode into a needle (Extended Data Fig. 1f). The method has demonstrated specificity for HNO, with no interference or spurious signal arising from •NO, O_2_, NO_2_^−^ and other RNS^11,13,31,32^. The calibration curve measures current responses at a potential of 0.8 V with the addition of freshly prepared AS, the HNO donor (Merck)^3,14,33^ and aqueous solutions of 30–900 nM nitroxyl (Extended Data Fig. 1c,d).

Electrochemical monitoring of HNO generation in vivo used a setup (microelectrode) previously described by Floryszak-Wieczorek et al.^34,35^ for •NO electrodetection in plant leaves. Briefly, a leaf blade was placed on an agar layer in which a Pt wire was introduced as a counter electrode. An AgCl-coated Ag needle was then introduced into the leaf tissue close to the area of HNO monitoring to serve as a quasi-reference electrode, and finally the HNO-selective microelectrode was introduced into the leaf.

### HNO quantification by a triarylphosphine-based probe

#### Confocal laser scanning microscopy

Cross-sections taken from the middle of the fully developed seventh leaf of the rosette were incubated in 100 µl of a 16 µM phosphine-based fluorescent probe (synthesized in S. B. King’s laboratory as described previously^17^) in 10 mM Tris–HCl, pH 7.4, for 15 min in the dark at 25 °C. As verified previously, the probe reacts with HNO under physiological conditions without interference by other biological redox species^36^. After incubation, the buffer was removed, and cross-sections were washed three times with 10 mM Tris–HCl, pH 7.4. Sections were placed on glass slides and observed under a Zeiss Axiovert 200M inverted microscope equipped with a confocal laser scanner (Zeiss LSM 510, Carl Zeiss AG). Sections were excited at 488 nm using an argon laser. Dye emissions were recorded using a 505–530 nm bandpass filter, and chloroplast autofluorescence was captured with the 585 nm long-pass filter. Microscope, laser and photomultiplier settings were held constant to obtain comparable data. Images were processed and analysed using Zeiss LSM 510 software (v.3.2 SP2).

A portion of *Arabidopsis* cell culture (250 μl) was incubated with a 16 µM phosphine-based fluorescent probe in 10 mM Tris–HCl, pH 7.4 for 15 min in the dark at 25 °C, then centrifuged at 2,000*g*, washed twice in fresh Tris–HCl buffer, and immediately imaged by confocal laser scanning microscopy as described for leaf cross-sections.

#### Fluorescence quantification

To measure the amount of HNO produced by *Arabidopsis*, 250 μl of cell culture sample or ten discs of 0.5 cm diameter cut from the middle of the fully developed seventh leaf were incubated in buffer containing 16 µM of phosphine-based fluorescent probe in 10 mM Tris–HCl, pH 7.4, for 1 h in darkness at 25 °C. Next, the discs were washed twice with 10 mM Tris–HCl, pH 7.4, finely homogenized in 1 ml Tris–HCl buffer and centrifuged at 900*g* at room temperature. Fluorescence was measured at 465 nm excitation and 520 nm emission wavelength (Fluorescence Spectrophotometer F-2500, Hitachi). Samples were normalized to the recorded autofluorescence of the plant material incubated without the fluorescent dye.

### •NO quantification by electrochemical method

•NO generation in leaf tissues was monitored by constant potential amperometry with a NO-selective disc-type electrode^34,35,37–40^. The electrode was prepared by electropolymerizing a poly-eugenol thin film on a cleaned Pt disc^37^ and repeatedly scanning the potential between −0.2 and 0.6 V in a 10 mM solution of eugenol (Merck) in 0.1 M NaOH. The modified electrode was then conditioned at a constant potential of 0.9 V in a phosphate buffer (pH 7.4) until a stable background current was reached. Electrochemical monitoring of NO generation in leaf tissue extracts and cell suspensions was performed as described previously^37^. The current was recalculated into concentration units based on a calibration curve (ISO-NO Mark II instruction manual, World Precision Instruments) constructed by measuring current responses to the addition of freshly prepared •NO aqueous solutions generated in situ from the reaction of iodide with nitrite in acid solution within the range of 0.3–100 μM (Extended Data Fig. 1g,h; ISO-NO Mark II instruction manual, World Precision Instruments)^41^.

### Crude leaf extract preparation for electrochemical measurements

Electrochemical monitoring of HNO and •NO generation in *Arabidopsis* leaf tissues was performed following Floryszak-Wieczorek et al.^34^ for •NO electrodetection in plant leaves. Briefly, leaves five to seven were pooled from different plants to obtain 0.5 g of fresh weight and homogenized under limited access to oxygen (in a hypoxia chamber; HypoxyLab) in 0.5 ml of 0.05 M phosphate buffer, pH 7.4, at 4 °C. The extract was centrifuged at 900*g* for 15 s at 4 °C and analysed immediately. All data were obtained following the same protocol, and the results were normalized to the same time.

### Quantification of chlorophyll contents

Chlorophyll was extracted from leaf seven (100 mg fresh material), following Hiscox and Israelstam^42^, incubated with 5 ml of dimethylsulfoxide (Merck) at 65 °C for 2 h. Chlorophyll *a* content was measured by spectrophotometer (Shimadzu UV-Vis-160) with emission at 665 nm and chlorophyll *b* content was measured at 649 nm.

### Pharmacological modulation of the cellular redox environment

To shift the cellular redox balance toward reduction, leaves of 21-day-old WT *Arabidopsis* plants were sprayed with 1 mM NAC (Merck), which depletes oxidized electron acceptors, such as glutathione and thioredoxin^43^, to induce reductive stress. To shift the balance toward oxidation, leaves of 21-day-old WT *Arabidopsis* plants were sprayed with 100 μM MN (Merck), a redox-active quinone that generates intracellular superoxide^44^. To identify HNO sources under reductive environment leaves of 21-day-old WT *Arabidopsis* plants were sprayed with 1 mM ascorbic acid (Merck), 1 mM salicylic acid (Merck) and 1 mM sodium hydrosulfide (as a H_2_S donor, Merck). In all treatments, control plants were sprayed with distilled water. Analyses were performed 0, 1, 3, 6 and 24 h after treatment (in the case of H_2_S, HNO formation was also monitored over a period of 1 h).

### Determination of redox status markers

#### ROS measurement

The O_2_^•−^ level was assayed spectrophotometrically based on the capacity of the superoxide anion-radical to reduce nitro blue tetrazolium (Merck) to diformazan^45^. H_2_O_2_ concentration was assayed spectrophotometrically using the titanium (Ti^4+^) method^46^.

#### Total antioxidant activity

Following Arts et al.^47^, we calculated Trolox Equivalent Antioxidant Capacity based on scavenging of the 2,2′-azinobis-(3-ethylbenzothiazoline-6-sulfonic acid) radical (ABTS^•^; Merck), which converts it into a colourless product.

#### Glutathione

Reduced glutathione (GSH) and oxidized glutathione (GSSH) contents were determined as described by Griffith^48^. GSH was oxidized by 5,5′-dithiobis-(2-nitrobenzoic acid) (Merck) to form GSSH and 5-thio-2-nitrobenzene. GSSH was then reduced to GSH by glutathione reductase and NADPH. Leaf tissues (200 mg), ground with a mortar and pestle in liquid nitrogen, were centrifuged at 15,000*g* for 15 min at 4 °C with 2.5 ml of 2.5% trichloroacetic acid. A supernatant (0.3 ml) was then used to assay total glutathione (GSH + GSSH). A further 0.3 ml of supernatant was pretreated with 6 μl of 2-vinylpyridine (Merck) for 60 min at 20 °C to mask GSH by derivatization. Both types of sample (0.1 ml each) were mixed with 0.7 ml of 0.3 mM NADPH, 0.1 ml of 6 mM 5,5′-dithiobis-(2-nitrobenzoic acid) and 0.1 ml of glutathione reductase (50 units ml^−1^). Absorbance at 412 nm was recorded after 5 min at room temperature. The total glutathione (GSH + GSSH) and GSSH contents were calculated using a standard curve and expressed as μmol per g (fresh weight). GSH content was calculated from the difference between total glutathione and GSSH.

### Cell viability determination

Cell viability, defined by plasma membrane integrity, was measured spectrophotometrically as Evans blue uptake^49^.

### Gene expression analysis

*Arabidopsis* leaves were frozen in liquid nitrogen and stored at −80 °C until use. RNA was isolated from 100 mg of a frozen sample using TriReagent (Merck) and purified using a Deoxyribonuclease Kit (Merck). We processed 1 µg of RNA for reverse transcription using a Reverse Transcription Kit (Thermo Fisher Scientific) following the manufacturer’s instructions. Real-time PCR was performed on a QuantStudio 3 thermocycler (Thermo Fisher Scientific) with QuantStudio Design and Analysis software (v.1.5.0). The reaction mixture contained 0.1 µM of each primer (Supplementary Table 5), 1 µl of 5× diluted complementary DNA, 5 µl of Power SYBR Green PCR Master Mix (Thermo Fisher Scientific) and diethyl pyrocarbonate-treated water to a total volume of 10 µl. PCR initiated denaturation at 95 °C for 5 min, then 50 cycles of 10 s at 95 °C, 20 s at 56 °C and 30 s at 72 °C. The reaction was finalized by denaturation at temperatures increasing by 1 °C every 5 s from 72 °C to 95 °C. Reaction specificity and threshold cycle values for individual samples were determined using the real-time PCR Miner Program (v.4.0)^50^. The *Arabidopsis* *AKT2* (*actin2*) gene was selected as a reference. Supplementary Table 5 lists all the primers (Genomed) used. Relative gene expression was calculated using the Pfaffl mathematical model^51^.

### Read mapping and identification of differentially expressed genes

TXPTS-treated versus control (untreated) samples were analysed. The complementary DNA library for RNA sequencing was prepared using a standard TruSeq Stranded messenger RNA kit from Illumina. Sequencing was performed on an Illumina NovaSeq machine with paired-end setting and a 150-nucleotide read length. The quality of raw sequencing reads was analysed using FastQC (v.0.11.9) software (https://www.bioinformatics.babraham.ac.uk/projects/fastqc/). Next, reads were subjected to mapping to the reference genome of *Arabidopsis thaliana*, obtained from the Ensembl Plants database^52^, using RNA STAR (v.2.7.10a) software^53^. The gene expression quantification was obtained from the STAR aligner using ARAPORT11 gene annotation^54^ and subjected to differential expression analysis using the R (v.4.2.0) environment with the limma (v.3.52.0)^55^ and EdgeR (v.3.38.0)^56^ packages. Statistically significant differentially expressed genes were determined using an adjusted *P*-value cut-off of <0.05. Functional analysis of gene set enrichment was performed using g:Profiler web service (v. e105_eg52_p16_5d1f001)^57^.

### Statistical analysis

All included experiments were replicated three times on independently grown plants. In addition, each sample was tested in three technical repetitions, and results from representative data sets are presented. Statistical differences were calculated using two-tailed *t*-tests (*P* ≤ 0.05). Related information is listed in the source data.

### Reporting summary

Further information on research design is available in the Nature Portfolio Reporting Summary linked to this article.

## Supplementary information

Reporting Summary

Supplementary Table 1 Nitroxyl donors^66–74^.

Supplementary Table 2 The content of chlorophyll a and b in phenotypically different leaf zones undergoing DILS on day 5 (D5), and after 3-day recovery (R3).

Supplementary Table 3 Extended mechanisms for HNO formation.

Supplementary Table 4 Differentially expressed genes (TXPTS vs Control). Genes with adjusted *P* value <0.05 has been marked with bold.

Supplementary Table 5 Sequences of primers used for genotyping and RT–qPCR.

## Figures and Tables

**Extended Data Fig. 9:**
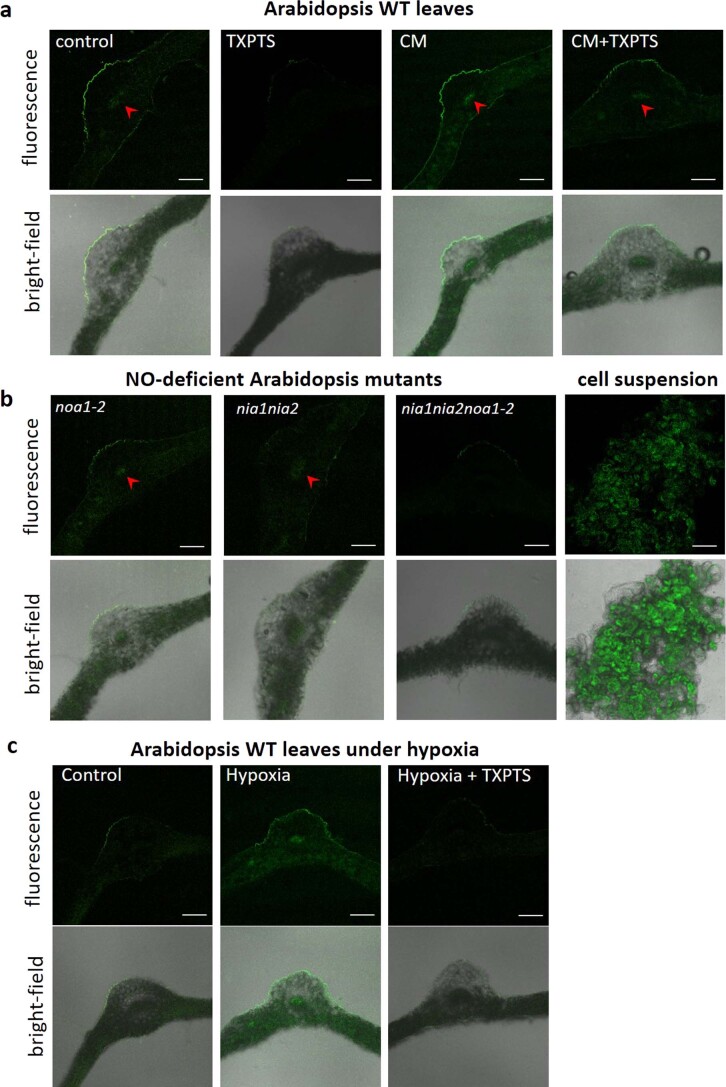
Visualization of HNO in Arabidopsis - fluorescence versus bright-field images. **a-c**, Representative confocal laser scanning fluorescence microscopy (top panel) and bright-field (bottom panel) images of a cross-section of Arabidopsis leaf or a cell suspension stained with 16 μM phosphine-based fluorescent probe (Ex: 488 nm, Em: 505–530 nm) in the absence (control) or presence of CM (1.5 mM), TXPTS (5 mM), or CM+ TXPTS, respectively. **a**, Arabidopsis WT leaves; **b**, *noa1-2, nia1nia2, nia1nia2noa1-2* – NO deficient mutants and cell suspension; **c**, Arabidopsis WT leaves at 48 h of hypoxia stress. Red arrows indicate vascular tissues; scale bars, 100 μm; cell suspension, 40 μm. For confocal observation the leaf cross-sections were randomly selected from ~ 20 slices pooled from leaves of different plants of the same genotype. The experiment was repeated independently three times with similar results.

## Data Availability

All data generated or analysed during this study are included in the manuscript or as a Supplementary Information (Extended Data Figs. 1–9 and Supplementary Tables 1–5). For RNA-seq data analysis, the *Arabidopsis thaliana* TAIR10 reference genome assembly has been used (GenBank ACC: GCA_000001735.1). RNA-seq data have been deposited in the European Nucleotide Archive (ENA) under accession number LPRJEB53633. Source data are provided with this paper.

## Source data

Source Data Fig. 1 Statistical source data.
Source Data Fig. 2 Statistical source data.
Source Data Fig. 3 Statistical source data.
Source Data Extended Data Fig. 1 Statistical source data.
Source Data Extended Data Fig. 2 Statistical source data.
Source Data Extended Data Fig. 3 Statistical source data.
Source Data Extended Data Fig. 5 Statistical source data.
Source Data Extended Data Fig. 6 Statistical source data.
Source Data Extended Data Fig. 7 Statistical source data.
Source Data Extended Data Fig. 8 Statistical source data.
