# Supplementary material for: Discovery of endogenous nitroxyl as a new redox player in Arabidopsis thaliana
Source: Nat Plants. 2022 Dec 23;9(1):36–44. doi: 10.1038/s41477-022-01301-z (PMC9873566; doi:10.1038/s41477-022-01301-z)
Supplement: Supplementary file 1 — Reporting Summary [file 41477_2022_1301_MOESM1_ESM.pdf]

nature portfolio

Corresponding author(s): Magdalena Arasimowicz-Jelonek

Last updated by author(s): Oct 20, 2022

Reporting Summary

Nature Portfolio wishes to improve the reproducibility of the work that we publish. This form provides structure for consistency and transparency in reporting. For further information on Nature Portfolio policies, see our Editorial Policies and the Editorial Policy Checklist.

Statistics

For all statistical analyses, confirm that the following items are present in the figure legend, table legend, main text, or Methods section.

n/a	Confirmed	
☐	☒	The exact sample size (*n*) for each experimental group/condition, given as a discrete number and unit of measurement
☐	☒	A statement on whether measurements were taken from distinct samples or whether the same sample was measured repeatedly
☐	☒	The statistical test(s) used AND whether they are one- or two-sided<br>*Only common tests should be described solely by name; describe more complex techniques in the Methods section.*
☒	☐	A description of all covariates tested
☐	☒	A description of any assumptions or corrections, such as tests of normality and adjustment for multiple comparisons
☐	☒	A full description of the statistical parameters including central tendency (e.g. means) or other basic estimates (e.g. regression coefficient) AND variation (e.g. standard deviation) or associated estimates of uncertainty (e.g. confidence intervals)
☐	☒	For null hypothesis testing, the test statistic (e.g. *F*, *t*, *r*) with confidence intervals, effect sizes, degrees of freedom and *P* value noted<br>*Give P values as exact values whenever suitable.*
☒	☐	For Bayesian analysis, information on the choice of priors and Markov chain Monte Carlo settings
☒	☐	For hierarchical and complex designs, identification of the appropriate level for tests and full reporting of outcomes
☒	☐	Estimates of effect sizes (e.g. Cohen's *d*, Pearson's *r*), indicating how they were calculated

Our web collection on statistics for biologists contains articles on many of the points above.

Software and code

Policy information about availability of computer code

Data collection	Data was collected exclusively using commercial softwares provided by technical equipment used for measurements (TEQ_HNO Software (v. 2.0), QuantStudio Design and Analysis software (v. 1.5.0), Zeiss LSM 510 software (v. 3.2 SP2)).
Data analysis	Data was analyzed using commercial or open source softwares (Real-time PCR Miner (v. 4.0) software, Zeiss LSM 510 software (v. 3.2 SP2)). The raw sequencing reads has been analyzed using FastQC (v. 0.11.9) software; reads were subjected to mapping to the reference genome of Arabidopsis thaliana, obtained from the Ensembl Plants database, using the RNA STAR (v. 2.7.10a) software. The gene expression quantification has been obtained from the STAR aligner using ARAPORT11 gene annotation and has been subjected to differential expression analysis using the R (v. 4.2.0) environment with limma (v. 3.52.0) and EdgeR (v. 3.38.0) packages. The functional analysis of gene set enrichment was performed using g:Profiler web service (v. e105_eg52_p16_5d1f001).

For manuscripts utilizing custom algorithms or software that are central to the research but not yet described in published literature, software must be made available to editors and reviewers. We strongly encourage code deposition in a community repository (e.g. GitHub). See the Nature Portfolio guidelines for submitting code & software for further information.

Data

Policy information about availability of data

All manuscripts must include a data availability statement. This statement should provide the following information, where applicable:

- Accession codes, unique identifiers, or web links for publicly available datasets
- A description of any restrictions on data availability
- For clinical datasets or third party data, please ensure that the statement adheres to our policy

The authore declare that all data supporting the findings of this study are available within the article and its Supplementary Information Files. For RNA-seq data analysis, the Arabidopsisn thaliana TAIR10 reference genome assembly has been used (GenBank ACC: GCA_000001735.1). RNA-seq data that support the findings of this study has been deposited in the European Nucleotide Archive (ENA) under accession code LPRJEB53633. Materials generated in this study are available from the corresponding author upon request.

Human research participants

Policy information about studies involving human research participants and Sex and Gender in Research.

Reporting on sex and gender	N/A
Population characteristics	N/A
Recruitment	N/A
Ethics oversight	N/A

Note that full information on the approval of the study protocol must also be provided in the manuscript.

Field-specific reporting

Please select the one below that is the best fit for your research. If you are not sure, read the appropriate sections before making your selection.

☒ Life sciences ☐ Behavioural & social sciences ☐ Ecological, evolutionary & environmental sciences

For a reference copy of the document with all sections, see nature.com/documents/nr-reporting-summary-flat.pdf

Life sciences study design

All studies must disclose on these points even when the disclosure is negative.

Sample size	Sample size per biological replicate (per treatment) was at least 25 plants which made it possible to obtain fresh weight of leaves necessary for molecular and biochemical analyzes. All included experiments were replicated three times (n=3). Sample size used in experiments was sufficient to generate statistical significance.
Data exclusions	No data was excluded from the analysis.
Replication	All included experiments were replicated three times on independently grown (and treated) plants. Additionally, each sample was tested in three technical repetitions. The number of replication is indicated in the figure legends. Similar results were obtained between independent experiments.
Randomization	Plants were always randomly distributed during growth and treatment.<br>For the preparation of a leaf sample, leaves were pooled from randomly selected different plants of the same genotype and treatment to obtain fresh weight appropriate to the experiment.<br>For confocal observation, the leaf cross-sections were randomly selected from ~ 20 slices pooled from leaves of randomly selected different plants of the same genotype and treatment.
Blinding	Blinding was not used in this study as our study did not involve animals and/or human research participants. All the experiments were performed without prior knowledge of the outcome.

Reporting for specific materials, systems and methods

We require information from authors about some types of materials, experimental systems and methods used in many studies. Here, indicate whether each material, system or method listed is relevant to your study. If you are not sure if a list item applies to your research, read the appropriate section before selecting a response.

Materials & experimental systems

n/a	Involved in the study
☒	☐ Antibodies
☐	☒ Eukaryotic cell lines
☒	☐ Palaeontology and archaeology
☒	☐ Animals and other organisms
☒	☐ Clinical data
☒	☐ Dual use research of concern

Methods

n/a	Involved in the study
☒	☐ ChIP-seq
☒	☐ Flow cytometry
☒	☐ MRI-based neuroimaging

Eukaryotic cell lines

Policy information about cell lines and Sex and Gender in Research

Cell line source(s)	Arabidopsis thaliana (Col-0) cell suspension culture from leaf-derived callus was grown following the protocol Encina et al. (2001).
Authentication	*Describe the authentication procedures for each cell line used OR declare that none of the cell lines used were authenticated.*
Mycoplasma contamination	*Confirm that all cell lines tested negative for mycoplasma contamination OR describe the results of the testing for mycoplasma contamination OR declare that the cell lines were not tested for mycoplasma contamination.*
Commonly misidentified lines (See ICLAC register)	*Name any commonly misidentified cell lines used in the study and provide a rationale for their use.*
