# Supplementary material for: Discovery of endogenous nitroxyl as a new redox player in Arabidopsis thaliana
Source: Nat Plants. 2022 Dec 23;9(1):36–44. doi: 10.1038/s41477-022-01301-z (PMC9873566; doi:10.1038/s41477-022-01301-z)
Supplement: Supplementary file 2 — Nitroxyl donors66–74. [file 41477_2022_1301_MOESM2_ESM.docx]

**Supplementary Table 1.** Nitroxyl donors.

| **Name** | **Short name** | **Structure** | **Characteristic** | **Half-live at 25℃ (min)** | **Starts pH HNO donation** | **Ref.** |
| --- | --- | --- | --- | --- | --- | --- |
| **Angeli´s Salt** | AS | 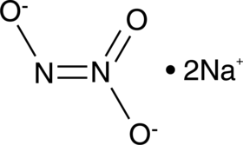 | Sodium trioxodinitrate(II) monohydrate | 16 | 4.0 ± 0.3 | ^66–68^ |
| **4-nitro Piloty´s Acid** | NPA | 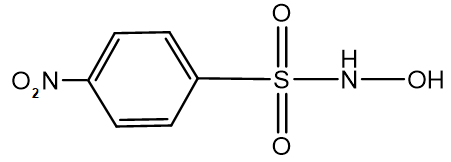 | Derived from N-hydroxybenzenesulfonamide (C_6_H_5_SO_2_NHOH, Piloty’s acid, PA) | 32 | 5.7 ± 0.8 | ^14,15,69–73^ |
| **Cimlanod**  **(BMS-986231,**  **CXL-1427)** | CM | 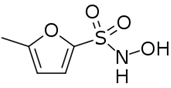 | Second-generation HNO donor | 40-144 | neutral pH | ^16,74^ |
