# Supplementary material for: Discovery of endogenous nitroxyl as a new redox player in Arabidopsis thaliana
Source: Nat Plants. 2022 Dec 23;9(1):36–44. doi: 10.1038/s41477-022-01301-z (PMC9873566; doi:10.1038/s41477-022-01301-z)
Supplement: Supplementary file 3 — The content of chlorophyll a and b in phenotypically different leaf zones undergoing DILS on day 5 (D5), and after 3-day recovery (R3). [file 41477_2022_1301_MOESM3_ESM.docx]

**Supplementary Table 2.** The content of chlorophyll *a* and *b* in phenotypically different leaf zones undergoing DILS on day 5 (D5), and after 3-day recovery (R3).

|  | **chl *a***  **µg x g^-1^ FW** | **chl *b***  **µg x g^-1^ FW** |
| --- | --- | --- |
| **zone a (control D5)**  *(see Fig. 3B)* | 0.787 ± 0.058 | 0.2590 ± 0.004 |
| **zone b (DILS D5)**  *(see Fig. 3B)* | 0.278 ± 0.054 | 0.0864 ± 0.004 |
| **zone c (DILS D5)**  *(see Fig. 3B)* | 0.109 ± 0.032 | 0.0345 ± 0.003 |
| **Control D3** | 0.765 ± 0.062 | 0.242 ± 0.005 |
| **DILS D3** | 0.423 ± 0.055 | 0.141 ± 0.002 |
| **Recovery R3** | 0.546 ± 0.046 | 0.180 ± 0.004 |
