# Supplementary material for: Discovery of endogenous nitroxyl as a new redox player in Arabidopsis thaliana
Source: Nat Plants. 2022 Dec 23;9(1):36–44. doi: 10.1038/s41477-022-01301-z (PMC9873566; doi:10.1038/s41477-022-01301-z)
Supplement: Supplementary file 4 — Extended mechanisms for HNO formation. [file 41477_2022_1301_MOESM4_ESM.docx]

**Supplementary Table 3.** Extended mechanisms for HNO formation.

| Proposed mechanism for the reaction of NO^•^ with: | **Ref.** |
| --- | --- |
| Aromatic alcohols: upper panel - initial step of proton-coupled nucleophilic attack (PCNA) shown by the simultaneous nucleophilic attack (N.A) and proton transfer (P.T.).  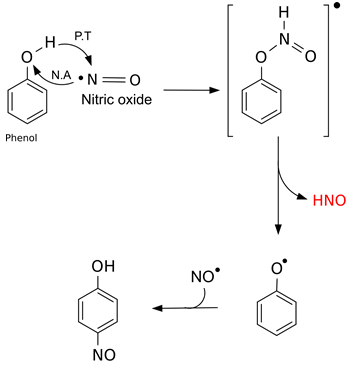 | ^63,64^ |
| Thiols  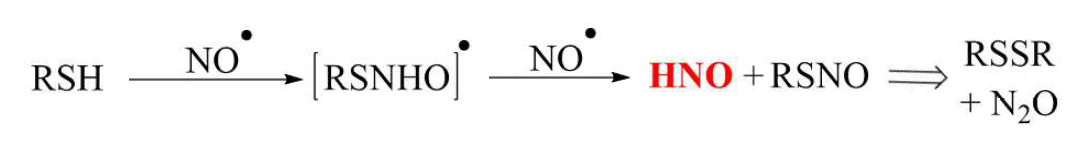 | ^29^ |
| H_2_S/HS^¯^: Large network of coupled reactions between H_2_S and NO• give rise to nitroxyl (HNO).  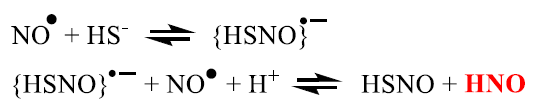 | ^4,65^ |
