# Supplementary material for: Discovery of endogenous nitroxyl as a new redox player in Arabidopsis thaliana
Source: Nat Plants. 2022 Dec 23;9(1):36–44. doi: 10.1038/s41477-022-01301-z (PMC9873566; doi:10.1038/s41477-022-01301-z)
Supplement: Supplementary file 5 — Differentially expressed genes (TXPTS vs Control). Genes with adjusted P value <0.05 has been marked with bold. [file 41477_2022_1301_MOESM5_ESM.docx]

**Supplementary Table 4**. Differentially expressed genes (TXPTS vs Control). Genes with adjusted p-value < 0.05 has been marked with bold.

| **Gene** | **Log_2_FC** | **Average expression** | **P-value** | **Adjusted  P-value** | **Gene symbol** | **Gene description** |
| --- | --- | --- | --- | --- | --- | --- |
| **AT5G25350** | -2.4988 | 5.1221 | 2.73E-07 | 0.0050 | EBF2 | EIN3-binding F-box protein 2 |
| **AT1G04310** | -2.9975 | 0.5819 | 1.70E-06 | 0.0157 | ERS2 | Ethylene response sensor 2 |
| AT5G57760 | -3.8264 | -1.1639 | 1.02E-04 | 0.6293 | - | - |
| AT3G48360 | -3.3004 | 2.2780 | 1.10E-03 | 1.0000 | BT2 | BTB/POZ and TAZ domain-containing protein 2 |
| AT2G20670 | -1.7432 | 6.9457 | 1.32E-03 | 1.0000 | - | Expressed protein |
| AT1G19350 | -1.7048 | 6.3143 | 1.84E-03 | 1.0000 | BES1 | Brassinosteroid signaling positive regulator (BZR1) family protein |
| AT1G73830 | -2.2072 | 2.0961 | 2.53E-03 | 1.0000 | BEE3 | Transcription factor BEE 3 |
| AT5G02540 | -2.4452 | 2.0302 | 2.77E-03 | 1.0000 | - | NAD(P)-binding Rossmann-fold superfamily protein |
| AT4G14930 | -1.5713 | 4.7155 | 2.97E-03 | 1.0000 | - | - |
| AT5G46330 | -1.9073 | 4.0142 | 4.30E-03 | 1.0000 | FLS2 | Leucine-rich repeat receptor-like protein kinase (Fragment) |
| AT3G23150 | -1.9813 | 0.3957 | 5.65E-03 | 1.0000 | ETR2 | Ethylene receptor 2, Involved in ethylene perception in Arabidopsis, mRNA is cell-to-cell mobile |
| AT5G56550 | -1.9532 | 4.4792 | 7.04E-03 | 1.0000 | OXS3 | Oxidative stress 3, involved in tolerance to heavy metals and oxidative stress |
| AT5G23240 | 2.5173 | 4.1324 | 7.23E-03 | 1.0000 | DJC76 | Chaperone protein dnaJ C76, chloroplastic |
| AT1G49210 | -3.4016 | -1.4695 | 8.45E-03 | 1.0000 | ATL76 | E3 ubiquitin-protein ligase ATL76 |
| AT5G21940 | -1.7094 | 6.4345 | 9.93E-03 | 1.0000 | - | - |
| AT2G44080 | -2.0420 | 1.7751 | 1.03E-02 | 1.0000 | ARL | ARGOS-like protein |
| AT4G37610 | -2.0414 | 4.4563 | 1.38E-02 | 1.0000 | BT5 | BTB/POZ and TAZ domain-containing protein 5 |
| AT1G06080 | -2.7067 | -0.4910 | 1.40E-02 | 1.0000 | ADS1 | Delta-9 acyl-lipid desaturase 1 |
| AT5G55250 | -1.7680 | 1.2127 | 1.45E-02 | 1.0000 | IAMT1 | Indole-3-acetate O-methyltransferase 1 |
| AT5G19120 | -1.4723 | 5.8916 | 1.54E-02 | 1.0000 | - | T24G5_20, Eukaryotic aspartyl protease family protein |
| AT2G43870 | -3.9000 | -3.4166 | 1.62E-02 | 1.0000 | - | Pectin lyase-like superfamily protein |
| AT4G10910 | -3.1651 | -1.2325 | 1.62E-02 | 1.0000 | -- | Uncharacterized protein At4g10910/F25I24_120 |
| AT4G16260 | 3.2134 | 1.8634 | 1.63E-02 | 1.0000 |  | Probable glucan endo-1,3-beta-glucosidase At4g16260 |
| AT5G50335 | -2.0076 | 0.9514 | 1.64E-02 | 1.0000 | - | - |
| AT1G18400 | -1.8947 | 1.6084 | 1.67E-02 | 1.0000 | BEE1 | Transcription factor BEE 1 |
| AT3G49940 | -1.6429 | 4.2344 | 2.00E-02 | 1.0000 | LBD38 | LOB domain-containing protein 38 |
| AT4G11521 | -1.4932 | 2.0949 | 2.63E-02 | 1.0000 | CRK34 | Putative cysteine-rich receptor-like protein kinase 34 |
| AT4G27260 | -1.9922 | 2.0587 | 2.92E-02 | 1.0000 | GH3.5 | Indole-3-acetic acid-amido synthetase GH3.5 |
| AT3G59480 | 2.3081 | -0.1966 | 3.18E-02 | 1.0000 | - | Probable fructokinase-4 |
| AT1G14600 | -1.4159 | 2.2242 | 3.19E-02 | 1.0000 | - | Putative Myb family transcription factor At1g14600 |
| AT4G36850 | -1.7776 | 3.6741 | 3.33E-02 | 1.0000 | - | PQ-loop repeat family protein / transmembrane family protein |
| AT5G03670 | -2.3423 | -0.5117 | 4.15E-02 | 1.0000 | TRM28 | histone-lysine N-methyltransferase SETD1B-like protein |
| AT1G53170 | -1.3148 | 3.4015 | 4.52E-02 | 1.0000 | ERF8 | Ethylene-responsive transcription factor 8 |
| AT4G16740 | 2.9933 | -0.4638 | 4.58E-02 | 1.0000 | TPS03 | Tricyclene synthase, chloroplastic |
| AT1G56300 | 1.5854 | 4.0084 | 4.67E-02 | 1.0000 | - | - |
| AT4G38650 | -1.8235 | 0.2644 | 4.95E-02 | 1.0000 | - | - |
