# Supplementary material for: Discovery of endogenous nitroxyl as a new redox player in Arabidopsis thaliana
Source: Nat Plants. 2022 Dec 23;9(1):36–44. doi: 10.1038/s41477-022-01301-z (PMC9873566; doi:10.1038/s41477-022-01301-z)
Supplement: Supplementary file 6 — Sequences of primers used for genotyping and RT–qPCR. [file 41477_2022_1301_MOESM6_ESM.docx]

**Supplementary Table 5.** Sequences of primers used for genotyping and RT-qPCR.

| **Gene name (locus)** | **Primer name** | **Sequences of the primer (5’ → 3’)** | **Restriction enzyme** | **Approach** |
| --- | --- | --- | --- | --- |
| *NOA1* (At3g47450) | AtNOA1.1-LP^26^ | GCACCTACACCACAGGCAAGC |  | *noa1-2* genotyping |
|  | AtNOA1.1-RP^26^ | CCAATTGGCAATGTTGGTCG |  |  |
| T-DNA (left border) | SAIL LB3^26^ | TAGCATCTGAATTTCATAACCAATCTCGATACAC |  |  |
| *NIA1* (At1g77760) | CAPS NR1-F^26^ | TACGACGACTCCTCAAGCGAC | HhaI | *nia1* genotyping |
|  | CAPS NR1-R^26^ | GGCTATAGATCCCGCATCGAC |  |  |
| *NIA2 (*At1g37130) | NR2.1-LP^26^ | ACGGCGTGGTTCGTTCTTACA |  | *nia2* genotyping |
|  | NR2.1-RP^26^ | ACCTTCTTCGTCGGCGAGTTC |  |  |
| *SAG12* (At5g45890) | SAG12-F^21^ | GGCGTTTTCAGCGGTTGCGG |  | RT-qPCR |
|  | SAG12-R^21^ | CCGCCTTCGCAGCCAAAATCG |  |  |
| *SAG20* (At3g10985) | SAG20-F^21^ | TCGGTAACGTTGTTGCTGGA |  | RT-qPCR |
|  | SAG20-R^21^ | ACCAAACTCTTTCAAATCGCCA |  |  |
| *SEN4* (At4g30270) | SEN4-F^21^ | GACTCTTCTCGTGGCGGCGT |  | RT-qPCR |
|  | SEN4-R^21^ | CCCACGGCCATTTCCCCAAGC |  |  |
| *EBF2* (At5g25350) | EBF2_F | GGTTTAGGTTCGTGATAGTGCC |  | RT-qPCR |
|  | EBF2_R | CCGAAGAGTTGTAATGGCGG |  |  |
| *ERS2* (At1g04310) | ERS2_F | AGCTTGATCGTGAGGTTGGG |  | RT-qPCR |
|  | ERS2_R | TTGGACAACTCCACAAGCGT |  |  |
| *ACT2* (At3g18780) | qACT2-F^21^ | TTGTTCCAGCCCTCGTTTGT |  | RT-qPCR normalisation |
|  | qACT2-R^21^ | TGTCTCGTGGATTCCAGCAG |  |  |
